# Innocent until Primed: Mock Jurors' Racially Biased Response to the Presumption of Innocence

**DOI:** 10.1371/journal.pone.0092365

**Published:** 2014-03-18

**Authors:** Danielle M. Young, Justin D. Levinson, Scott Sinnett

**Affiliations:** 1 Department of Psychology, Rutgers University, New Brunswick, New Jersey, United States of America; 2 William S. Richardson School of Law, University of Hawaii at Manoa, Honolulu, Hawaii, United States of America; 3 Department of Psychology, University of Hawaii at Manoa, Honolulu, Hawaii, United States of America; CSIC-Univ Miguel Hernandez, Spain

## Abstract

**Background:**

Research has shown that crime concepts can activate attentional bias to Black faces. This study investigates the possibility that some legal concepts hold similar implicit racial cues. Presumption of innocence instructions, a core legal principle specifically designed to eliminate bias, may instead serve as an implicit racial cue resulting in attentional bias.

**Methodology/Principal findings:**

The experiment was conducted in a courtroom with participants seated in the jury box. Participants first watched a video of a federal judge reading jury instructions that contained presumption of innocence instructions, or matched length alternative instructions. Immediately following this video a dot-probe task was administered to assess the priming effect of the jury instructions. Presumption of innocence instructions, but not the alternative instructions, led to significantly faster response times to Black faces when compared with White faces.

**Conclusions/Significance:**

These findings suggest that the core principle designed to ensure fairness in the legal system actually primes attention for Black faces, indicating that this supposedly fundamental protection could trigger racial stereotypes.

## Introduction

In 2013, the acquittal of George Zimmerman, who was tried for the murder of Black teenager Trayvon Martin, reinvigorated questions about whether the criminal justice system treats all individuals, regardless of race, without bias. Multidisciplinary empirical evidence suggests that, indeed, the American criminal justice system is not unbiased. For example, one of eight Black males in their twenties is incarcerated at any particular moment, compared to just one of fifty-nine White males of the same age [1]. Furthermore, between 1930 and 1982 African Americans constituted 53% of those executed, with this imbalance only slightly improving since then [2]. While the social reasons behind these trends in the legal system are complex, research suggests that automatic activations of racial stereotypes might play a critical role in explaining racial disparities in the criminal justice system [3]. Specifically, research has shown that concepts of crime cue attention for the racial category of Black [4]. The current research investigates if these attentionally biasing racial cues are present in the legal system.

Research has documented the powerful consequences of conceptualizing Black men as aggressive criminals [5]. For instance, explicit shooting-game paradigms have demonstrated that people view Black targets as more threatening and are quicker to shoot armed Black targets than armed White targets [6], [7]. Even trained police officers take longer to make the decision to not shoot an unarmed Black suspect when compared with a comparable White suspect [8]. In the courtroom context, researchers have found that, when presented with a suspect from a stereotyped group, mock jurors view ambiguous pieces of evidence as indicating guilt [9], [10], and in the author's unpublished data are more likely to convict a Black than a White defendant of a more aggressive and intentional crime.

Consistent with the above results, merely cueing racial categories can also lead to biased behaviors in the criminal justice system. For example, priming with Black faces increases attention to crime-related objects, leading both to faster identification of guns and misidentification of tools as weapons [11]. Relatedly, using priming words that are semantically associated with the racial category of Black (i.e., *gospel, hood*, and *segregation*) has been shown to alter legal judgments of police officers, juvenile probation officers, and judges in racially biased ways [12], [13].

Interestingly, the relationship between the concepts of crime and Black appears to be bidirectional. Research demonstrates that, in addition to Black face primes increasing the speed of detecting weapons [4], priming the concept crime (using objects such as knives, guns, and police badges or words such as *stop, chase*, and *apprehend*) can increase attention to Black faces [4]. This research suggests that concepts such as crime, which are highly conceptually linked with a racial category, can serve as a prime for that category. Since the justice system is imbued with racial connections, it is important to explore if and how racial priming is present in other aspects of the justice system, and specifically in those areas of the law that seek to further justice and fairness.

The presumption of innocence, a foundation of every criminal trial designed to create a fair and just proceeding, presents as an excellent candidate to explore conceptual links between race and specific legal processes intended to promote fairness. In every criminal trial, the judge explains to the jury that the defendant must be considered innocent unless and until the prosecution proves beyond a reasonable doubt that the defendant is guilty [14]. These judicial instructions are the primary tools relied upon by the criminal justice system to ensure that an innocent defendant is not wrongfully convicted. Previous research suggests the presumption of innocence may not operate as intended. For example, people implicitly associate Black with guilt (and White with not guilty) [15]. Thus, despite explicit instructions that any individual must be presumed innocent until proven guilty, Black defendants may be implicitly associated with the construct of guilt that underlies the presumption of innocence before a trial even begins.

Considered together with the massive racial disparities that continue to plague the criminal justice system as a whole, it is possible that the fundamental legal protection of the presumption of innocence has been stripped of its protective meaning by a culture that associates Blacks with violence, crime, and guilt [4]. Thus, the very principle designed to create an impartial atmosphere might inadvertently prime attention to race. In other words, in the legal system, the presumption of innocence could actually prime racial constructs rather than ensuring a fair trial. Previous studies have only tested specific implicit associations between guilt and racial categories, and have not investigated what happens when the dichotomous concept of innocence and guilt is actually activated in a judicial setting, even prior to the case being heard.

## Current Study

The current study is the first of its kind to explore this potential racial prime (presumption of innocence) in the context of the legal setting, and complements current literature that explores how the criminal justice system might inadvertently prime race related stereotypes. A limitation of the existing racial priming research is that most studies, if not all, have been conducted in laboratory settings, which begs the question of if and how racial priming might occur in a more ecologically valid setting, especially one crucial to American society [16]. A single study investigating the impact of the courtroom setting on legal judgments found, for example, that the jury setting itself may have a priming effect on citizens called to jury service [17]. Participants who were told they were jurors in a trial (compared to those who were told they were reading facts from a newspaper article) made heightened attributions about causation, and more purposeful judgments of intentionality. No studies, however, have tested whether individual legal principles presented to a jury, such as the presumption of innocence, could have similar priming effects.

Although current evidence indicates that explicit racial cues (i.e., mentioning a Black defendant) can lead to changes in behavior and judgments in the legal setting [9], [10], [13], [18], little research has explored the role of implicit racial cues in this setting. In this context implicit does not mean the racial cue is presented below threshold, instead it denotes that an individual may be unaware of the racial cue embedded in a stimulus. Research from other domains has suggested that, at least in political communications, implicit racial cues (communicating about *inner city, anti-poverty reform*, and *disadvantaged teenagers* to conjure up racial images) exist, are frequently used, and alter perceptions [19], [20]. It is equally important to explore these possibilities in the legal arena.

The current research explores important new avenues. First, it investigates the possibility of implicit racial cues embedded in the legal system. Second, it explores this possibility in a courtroom setting, in order to improve the ecological validity of the findings. Thus, using presumption of innocence instructions, the linchpin of any criminal trial, we explored the possibility that attention to the racial category of Black can be primed in an extremely meaningful societal setting. This study investigates the hypothesis that the presumption of innocence has become associated with the social category of Black, in the same way that the link between the concepts of Black and athletes [21], and Black and apes [22], has been demonstrated through research.

## Methods

### Ethics statement

Written informed consent, abiding to and approved by the University of Hawaii at Manoa's ethics committee (Committee for Human Subjects (CHS)), was obtained prior to participating in the experiment. This study was approved by the University's CHS.

### Participants

Sixty-one jury eligible, ethnically diverse (24 identified as Asian-American, 16 as European-American, 21 as other) students (female *n* = 33, *m*_age_ = 19.80) participated in this study for course credit. This study was approved for use of human subjects by the University of Hawaii IRB, and informed consent was obtained prior to the beginning of the experiment.

### Materials

#### Video prime

A White male United States District Court judge, who was blind to our hypotheses, was videotaped reading a series of jury instructions that either included the presumption of innocence and accompanying reasonable doubt instructions (PI) or alternative matched-length instructions (No PI; see Appendix S1 for details on the PI instructions). These instructions also included or omitted a crime description (assault with a dangerous weapon; see Appendix S1 for details) counterbalanced to control for the possibility that certain crimes might prime attention for Black faces. Initial analysis demonstrated no significant differences between the included (547 ms) and omitted (555 ms) crime description, p>.10, so these categories were collapsed for the remaining analyses. The judge wore his judicial robes and was filmed sitting down in front of a white background. The jury instructions lasted approximately 2:20 s.

#### Visual stimuli

Four faces, two Black and two White, were selected from a previously constructed database. Faces were approximately 5×7.6 cms, looked straight at the camera, were not smiling, and were on a plain white background. These faces had been previously matched for attractiveness and stereotypicality [4], and individual faces within race did not influence attention latencies.

### Procedure

Participants sat in the jury box of a courtroom in the William S. Richardson School of Law at the University of Hawaii, which also serves as a courtroom used by visiting 9^th^ Circuit and other judges. In groups of up to six, participants were randomly assigned to a 2 (PI/No-PI condition) X 2 (Crime Description/No Crime Description) condition.

Immediately after watching the jury instructions, participants completed a computer-based dot-probe priming task [23], presented on laptops provided in the jury box, to assess response latency to Black versus White faces. This task has been used frequently in racial bias research to establish priming effects of related categories and attention [4], [22], [24]. The task consisted of 64 trials where pairs of faces, one White and one Black, were presented for 450 ms on either side of the screen (approximately 7.5 cms on either side of fixation). The different faces were randomly paired (presented in equal proportions) and could appear on either side of the screen. Immediately after the faces disappeared, a small faint grey dot (6.35 mm) appeared in equal frequency behind one of the faces, which were irrelevant to the task of detecting the dot. Participants indicated as quickly as possible the side of the screen (left or right) that the dot had appeared with a key press.

## Results

We performed a natural log transformation to reduce a positive skew in the detection latency data, and all analyses were performed on the transformed data. The means follow the same pattern, so for ease of interpretation, reporting of reaction times is in milliseconds.

We submitted the transformed latency means to a mixed 2 (between subjects: PI or no PI) X 2 (within subjects: Black or White face) ANOVA. There was no main effect for PI condition (*M_PI_* = 544.7 ms, *SE* = 23.42, *M_No PI_* = 572 ms, *SE* = 20.87, *p* = .27). There was a main effect for race, in that participants were generally faster to respond to dots preceded by Black (*M* = 552.5 ms, *SE* = 16.38) than White (*M* = 564.1 ms, *SE* = 15.37) faces, F(1,59) = 7.73, p = .007, η^2^_partia l_ = .12).

**Figure 1:**
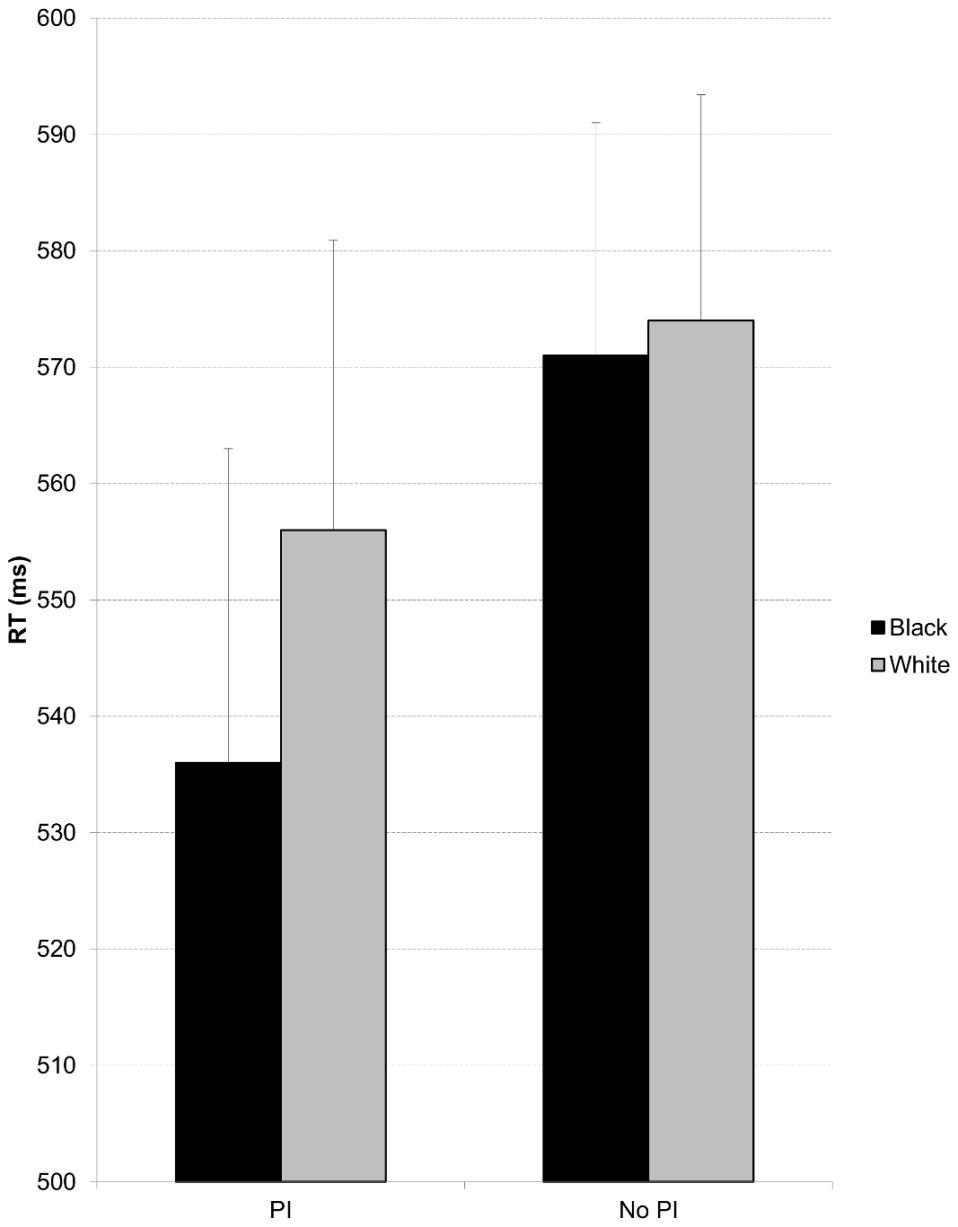
Attentional bias as a result of Presumption of Innocence Instructions. Mean detection latency as a function of prime (PI: Presumption of innocence instructions vs. No PI: no presumption of innocence instructions) and dot location (behind White or Black face). Error bars represent the average standard error for each condition.

This effect is qualified by the critical interaction of the race of face (Black or White) and PI condition (see Figure 1), *F*(1,59) = 4.43, *p = *.04, η^2^_partial_ = .07. Participants who received presumption of innocence instructions were significantly faster to identify a dot preceded by a Black face (*M* = 534.40 ms, *SE* = 24.50) than a White face (*M* = 555.04 ms, *SE* = 22.95), *F*(1,60) = 10.84, *p* = .002, while participants who did not receive PI instructions were equally fast to find the dot regardless of if it was preceded by Black or White face (570.72 ms vs 573.19 ms), F(1,60) = 0.22, *p* = .64.

## Discussion

Presumption of innocence instructions induced attentional bias. Specifically, individuals presented with presumption of innocence instructions had faster responses to Black, compared to White, faces in a dot-probe task. Participants who did not receive presumption of innocence instructions did not show this attentional bias. Thus, the presumption of innocence instructions lead to biased attention for Black faces suggesting that implicit racial cues are present in the judicial setting. It is important to emphasize this finding, as it establishes that racial attention can be primed simply by instructing mock jurors regarding the presumption of innocence. This attentional bias suggests that implicit racial cues, far from being a laboratory specific experience, are present in our legal system, and exist in a particularly counterintuitive and societally important place. That is, the very same instructions designed specifically to protect defendants from wrongful convictions, prime attention for Black faces in the same way that other categories have, such as stereotypically race related words (e.g., basketball or Harlem), and crime [4].

Presumption of innocence instructions shifted attention onto Black targets, but including a description of the crime committed, did not. There are several possible explanations for why we failed to observe this effect. Though previous studies have demonstrated that crime primes the category of Black [4], these studies were not set in a courtroom, a space that has been shown to alter the causal attributions of participants [17]. Furthermore, it is also possible that in the context of jury instructions that explicitly mention criminal proceedings, detailing a specific crime may have not been a greater prime than the jury instructions themselves. Though it is possible that this study lacks power to adequately address the impact of specifying the crime committed, the current analysis did not suggest a trend that might become significant given a larger sample size. Regardless, the presumption of innocence primed attention for Black faces, over and above the influence of a criminal proceeding.

A limitation of this study, and the overwhelming majority of research utilizing attentional priming, is that while it establishes that a realistic implicit racial cue can alter attention, it does not explore how such a prime scales up to observable behavior and decision-making. That is, despite Black being primed by presumption of innocence instructions, it is difficult to speculate what the consequences of this altered attention are. For instance, does this biased attention feed into the overrepresentation of Black Americans in the criminal justice system? Though it is reasonable to hypothesize that changes in behavior and judgment produced by laboratory primes will extend to more realistic situations, it is unwise to assume that this will be the case. It is therefore necessary to begin to establish the arenas where these primes exist, and to further explore how they affect behavior, judgments, and decision-making.

Since attentional priming is not inherently positive or negative, one could interestingly argue that the priming effect observed here is evidence that this set of jury instructions *advantages* Black individuals in a legal context. While the current experiment does not allow for a direct test of the positive or negative consequence of priming, converging evidence from other sources suggests that this attentional bias is likely negative. Attention to Black faces has been linked with threat perception [24] and, in legal settings, similar attention effects have promoted perceptions of guilt [25]. Indeed, activating the social category Black has been demonstrated to have negative consequences on legal judgments [12]. The effect of attention and perceptions of guilt is especially salient for minority groups [26], so priming attention to minority racial groups in a judicial setting is particularly troublesome. Similarly, because Black has been shown to be implicitly associated with Guilty, compared to White and Not Guilty, it would be surprising if this finding were completely reversed in the current research paradigm. Regardless, while the current findings suggest that the very principle designed to create an unbiased and impartial atmosphere inadvertently biases participants to the racial category of Black, more research is needed before one can explicitly claim that this would lead to increased negative consequences for the defendant.

This study also does not test for the specific mechanisms through which PI instructions primed for Black faces. However, it is possible to reasonably speculate on the underpinnings of this phenomenon. Previous research suggests that the concepts of guilt and Black are implicitly associated [15], and the definition of reasonable doubt (a vital portion of PI instructions) relies heavily upon the legal concept of guilt, and how guilt is determined in the legal context. Thus, the very use of the word “guilty” may have ironic consequences for defendants who are already implicitly associated with guilt. Understanding the exact mechanism that creates attentional bias, much like the downstream consequences of this bias, are vital next steps in understanding how unintentional bias may distort the legal system. More importantly, beyond illuminating racial cues in the judicial system, understanding how these racial cues impact legal processes can point to ways in which these biases may be attenuated.

Despite these limitations, this study presents a first look at implicit racial cues embedded in the legal system and demonstrates evidence for the presence of attentionally biasing cues in the legal system. Since simply being the subject of attention can alter how a suspect is perceived, including the eventual attribution of causality in both mundane and legal contexts [27], [28], the present findings are concerning for current judicial practices. Indeed, paired with an overall implicit association between Black and the legal concept of guilty, and White and not guilty [15], these systematic couplings of racial categories and aspects of the criminal justice system raise questions about the racial fairness of presumption of innocence instructions, a foundation of every criminal trial designed to create a fair and just proceeding. This suggests the possibility that the very instructions designed to protect defendants from bias may in fact activate implicit processes that produce biased responses.

## Supporting Information

Appendix S1 Jury Instructions. Instructions that were included *only* in the presumption of innocence condition are in **bold**. Instructions that were included *only* in the crime description condition are in *italics*. Matched instruction for the presumption of innocence and crime description conditions are indicated in [brackets].
(DOCX)
