# Supplementary material for: Innocent until Primed: Mock Jurors' Racially Biased Response to the Presumption of Innocence
Source: PLoS One. 2014 Mar 18;9(3):e92365. doi: 10.1371/journal.pone.0092365 (PMC3958515; doi:10.1371/journal.pone.0092365)
Supplement: Appendix S1 — Jury Instructions. Instructions that were included only in the presumption of innocence condition are in bold. Instructions that were included only in the crime description condition are in italics. Matched instruction for the presumption of innocence and crime description conditions are indicated in [brackets]. (DOCX) [file pone.0092365.s001.docx]

**Appendix S1. Jury Instructions.**

**DUTY OF JURY**

Ladies and gentlemen: You now are the jury in this case, and I want to take a few moments to tell you something about your duties as jurors and to give you some instructions. These are preliminary instructions. At the end of the trial I will give you more detailed instructions. Those instructions will control your deliberations.

You should not take anything I may say or do during the trial as indicating what I think of the evidence or what your verdict should be.

**THE CHARGE / PRESUMPTION OF INNOCENCE**

This is a criminal case brought by the United States government. *[The government charges the defendant with assault with a dangerous weapon.]*

The charge against the defendant is contained in the indictment.

*The indictment is simply the description of the charge made by the government against the defendant; it is not evidence of anything.*

*In order to help you follow the evidence, I will now give you a brief summary of the elements of the crime[s] which the government must prove to make its case:*

*First, the defendant assaulted the victim by intentionally wounding him using a display of force that reasonably caused him to fear immediate bodily harm;*

*Second, the defendant acted with the specific intent to do bodily harm to the victim; and*

*Third, the defendant used a dangerous weapon.*

These instructions are preliminary and the instructions I will give at the end of the case will control.

The defendant has pleaded not guilty to the charge **and is presumed innocent unless and until proved guilty beyond a reasonable doubt.** **A defendant has the right to remain silent and never has to prove innocence or present any evidence.**

[BENCH CONFERENCES AND RECESSES /matched instructions for the no crime description condition

From time to time during the trial, it may become necessary for me to talk with the attorneys out of the hearing of the jury, either by having a conference at the bench when the jury is present in the courtroom, or by calling a recess. Most often these conferences will involve determination as to whether evidence is admissible under the rules of evidence. It is appropriate to take these matters up outside the presence of the jury. Should I conclude that a more prolonged discussion is necessary, I may excuse you from the courtroom.]

**REASONABLE DOUBT—DEFINED**

**Proof beyond a reasonable doubt is proof that leaves you firmly convinced that the defendant is guilty. It is not required that the government prove guilt beyond all possible doubt.**

**A reasonable doubt is a doubt based upon reason and common sense and is not based purely on speculation. It may arise from a careful and impartial consideration of all the evidence, or from lack of evidence.**

**If after a careful and impartial consideration of all the evidence, you are not convinced beyond a reasonable doubt that the defendant is guilty, it is your duty to find the defendant not guilty. On the other hand, if after a careful and impartial consideration of all the evidence, you are convinced beyond a reasonable doubt that the defendant is guilty, it is your duty to find the defendant guilty.**

[RULING ON OBJECTIONS /matched instructions for the no presumption of innocence condition

There are rules of evidence which control what can be received into evidence. When a lawyer asks a question or offers an exhibit into evidence and a lawyer on the other side thinks that it is not permitted by the rules of evidence, that lawyer may object. If I overrule the objection, the question may be answered or the exhibit received. If I sustain the objection, the question cannot be answered, and the exhibit cannot be received. Whenever I sustain an objection to a question, you must ignore the question and must not guess what the answer would have been.

Sometimes I may order that evidence be stricken from the record and that you disregard or ignore the evidence. That means that when you are deciding the case, you must not consider the evidence which I told you to disregard.]

The trial will begin shortly.  Thank you in advance for your jury service.
